# Supplementary figures and images for: High-sensitivity calcium biosensor on the mitochondrial surface reveals that IP3R channels participate in the reticular Ca2+ leak towards mitochondria
Source: PLoS One. 2023 Jun 9;18(6):e0285670. doi: 10.1371/journal.pone.0285670 (PMC10256219; doi:10.1371/journal.pone.0285670)

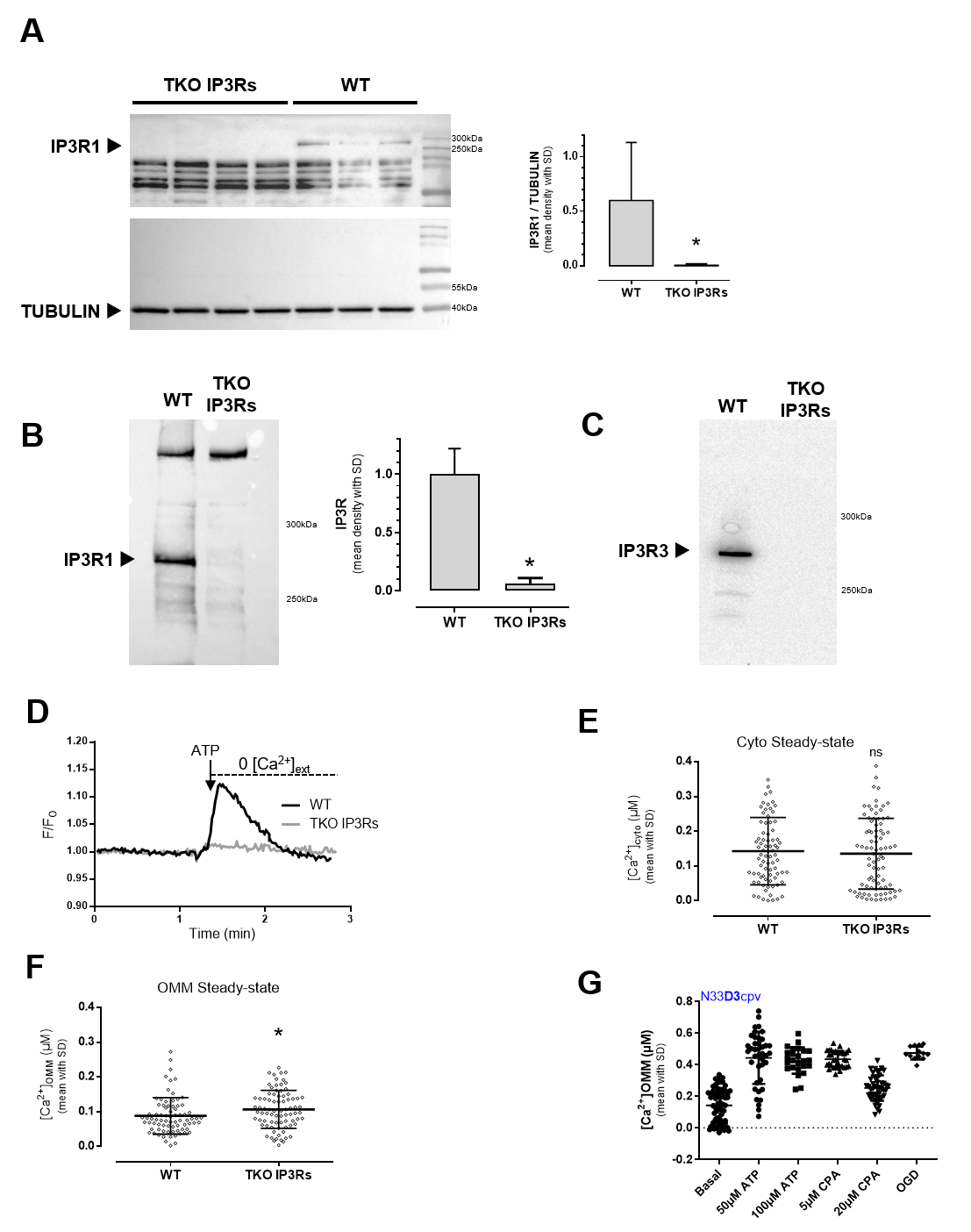

Supplement: S1 Fig — (A) Immunoblotting against IP3R1 isoform (IP3R-I Santa-Cruz sc-28614) and tubulin in WT and TKO IP3Rs HeLa cells. Data shown represent the mean with standard deviation (SD) of 3 independent experiments, (* p<0.05). (B) Immunoblotting against IP3R1 receptor (Anti-IP3 receptor antibody Abcam ab5804) in WT and TKO IP3Rs Hela cells. Proteins normalized by using 2,2,2-Trichloroethanol (TCE) to visualize total protein content. Data shown represent the mean with standard deviation (SD) of 3 independent experiments, (* p<0.05). (C) Immunoblotting against IP3R3 receptor (IP3R3 BD 610312, 1/1000, MOUSE) in WT and TKO IP3Rs Hela cells. Proteins normalized by using 2,2,2-Trichloroethanol (TCE) to visualize total protein content. (D) [Ca2+]OMM was estimated using N33D3cpv biosensor in WT and TKO IP3Rs HeLa cells treated with 100 μM Na, in absence of external Ca2+. Representative average FRET-ratio (F) normalized with the baseline FRET-ratio value (F0). (E) Steady-state [Ca2+]cyto in WT and TKO IP3Rs HeLa cells. (F) Steady-state [Ca2+]OMM in WT and TKO IP3Rs HeLa cells. The normality of the samples was evaluated (Kolmogorov-Smirnov test) and Mann–Whitney test (for non-normal distribution) was used. (G) [Ca2+]OMM steady state (basal) and peak measurements upon ATP, CPA and OGD treatment protocol with the N33D3cpv sensor. (TIF) [file pone.0285670.s001.tif]

Figure S1A

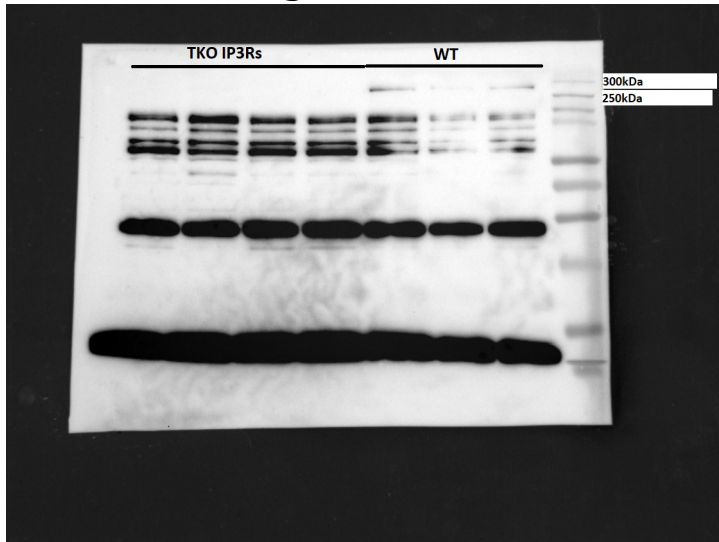

Figure S1A

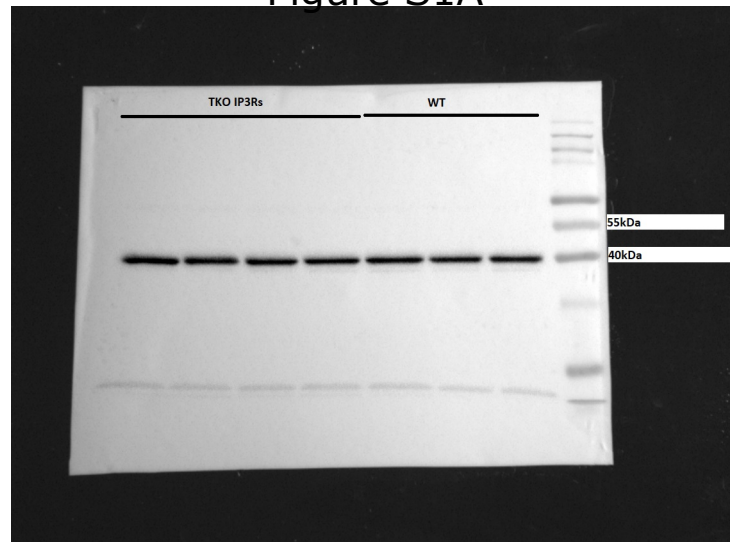

Figure S1B

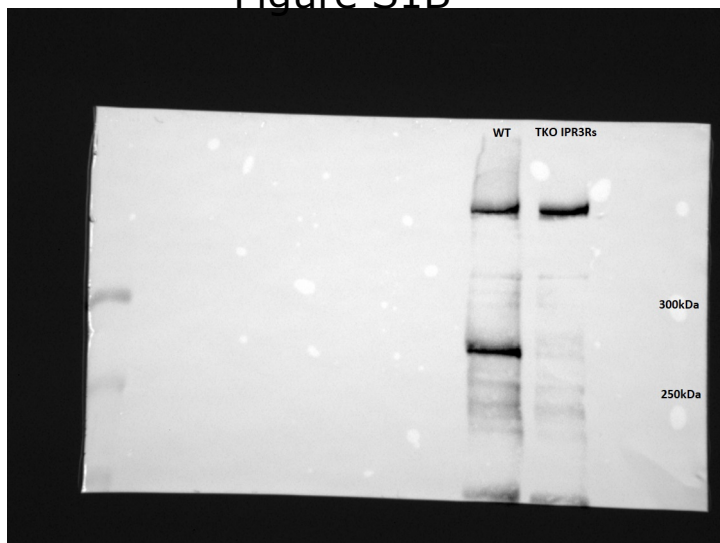

Figure S1C

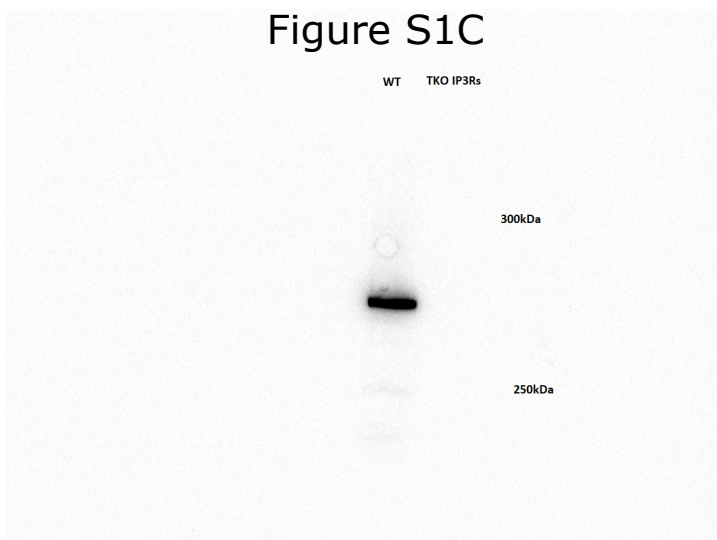

Supplement: S1 Raw images — (PDF) [file pone.0285670.s002.pdf]
